# Transgenic Mice Expressing Functional TCRs Specific to Cardiac Myhc-α 334–352 on Both CD4 and CD8 T Cells Are Resistant to the Development of Myocarditis on C57BL/6 Genetic Background

**DOI:** 10.3390/cells12192346

**Published:** 2023-09-25

**Authors:** Meghna Sur, Mahima T. Rasquinha, Rajkumar Arumugam, Chandirasegaran Massilamany, Arunkumar Gangaplara, Kiruthiga Mone, Ninaad Lasrado, Bharathi Yalaka, Aakash Doiphode, Channabasavaiah Gurumurthy, David Steffen, Jay Reddy

**Affiliations:** 1 School of Veterinary Medicine and Biomedical Sciences, University of Nebraska-Lincoln, Lincoln, NE 68583, USA; msur2@huskers.unl.edu (M.S.); mrasquinha2@huskers.unl.edu (M.T.R.); arrajkumar88@gmail.com (R.A.); mchandirasegaran@gmail.com (C.M.); arungb@gmail.com (A.G.); kmone2@huskers.unl.edu (K.M.); nlasrado@bidmc.harvard.edu (N.L.); bharathipuji@gmail.com (B.Y.); aakashdoiphode@gmail.com (A.D.); dsteffen1@unl.edu (D.S.); 2 Bristol Myers Squibb, Summit, NJ 07901, USA; 3 CRISPR Therapeutics, Boston, MA 02127, USA; 4 Miltenyi Biotec, Gaithersburg, MD 20878, USA; 5 Center for Virology and Vaccine Research, Harvard Medical School, Boston, MA 02115, USA; 6 Department of Animal Genetics and Breeding, Krantisinh Nana Patil College of Veterinary Science, Shirwal 412801, Maharashtra, India; 7 Department of Genetics, Cell Biology and Anatomy, University of Nebraska Medical Center, Omaha, NE 68198, USA; cgurumurthy@unmc.edu

**Keywords:** TCR transgenic mice, cardiac myosin-α 334–352, myocarditis, inflammatory cardiomyopathy

## Abstract

Myocarditis is a predominant cause of congestive heart failure and sudden death in children and young adolescents that can lead to dilated cardiomyopathy. Lymphocytic myocarditis mediated by T cells can result from the recognition of cardiac antigens that may involve CD4 or CD8 T cells or both. In this report, we describe the generation of T cell receptor (TCR) transgenic mice on a C57BL/6 genetic background specific to cardiac myosin heavy chain (Myhc)-α 334–352 and make the following observations: First, we verified that Myhc-α 334–352 was immunogenic in wild-type C57BL/6 mice and induced antigen-specific CD4 T cell responses despite being a poor binder of IA^b^; however, the immunized animals developed only mild myocarditis. Second, TCRs specific to Myhc-α 334–352 in transgenic mice were expressed in both CD4 and CD8 T cells, suggesting that the expression of epitope-specific TCR is common to both cell types. Third, although T cells from naïve transgenic mice did not respond to Myhc-α 334–352, both CD4 and CD8 T cells from animals immunized with Myhc-α 334–352 responded to the peptide, indicating that antigen priming is necessary to break tolerance. Fourth, although the transgenic T cells could produce significant amounts of interferon-γ and interleukin-17, the immunized animals developed only mild disease, indicating that other soluble factors might be necessary for developing severe myocarditis. Alternatively, the C57BL/6 genetic background might be a major contributing factor for resistance to the development of myocarditis. Taken together, our model permits the determination of the roles of both CD4 and CD8 T cells to understand the disease-resistance mechanisms of myocarditis in a single transgenic system antigen-specifically.

## 1. Introduction

Myocarditis refers to inflammation of the heart muscle layer. It is the third leading cause of cardiovascular death in young athletes, next only to coronary artery abnormalities and hypertrophic cardiomyopathy [1,2]. It is a predominant cause of heart failure in children and young adolescents and has been linked to sudden death in adults or young athletes in up to 12% of cases [3,4,5]. Reports indicate that 30% of myocarditis patients could develop dilated cardiomyopathy (DCM) [6,7]. Likewise, recent studies show that ~50% of clinical diagnoses of DCM involve immunohistochemically detectable myocarditis [8,9,10]. Clinically, DCM could be considered an end-stage disease [11]. Because there are no effective treatments, approximately half of DCM patients receive heart transplants, and children with acute myocarditis have only a 60% likelihood of transplantation-free survival at ten years post-diagnosis [12,13].

Histologically, the disease process can be classified into lymphocytic, neutrophilic, eosinophilic, granulomatous, and giant cell myocarditis [14]. Of these, lymphocytic myocarditis generally occurs with viral infections [15]. Although most myocarditis-affected individuals may spontaneously recover, up to 20% of them can develop DCM, in which autoimmunity is suspected as an underlying mechanism [6,7]. Although autoantibodies for various antigens—such as cardiac myosin (Myhc), adenine nucleotide translocator (ANT), branched-chain α-ketoacid dehydrogenase kinase (BCKDk), sarcoplasmic/endoplasmic reticulum Ca2^+^ adenosine triphosphatase 2a (SERCA2a), β1 adrenergic receptor (β1AR), muscarinic M2 acetylcholine receptor, mitochondrial M7, and cardiac troponin I (cTNI)―have been detected in DCM patients [16,17,18,19,20,21,22,23,24,25], their role remains unclear, and isolated reports are available as to the role T cells play in DCM’s pathogenesis [26]. To address autoimmune events, various autoimmune myocarditis models have been developed since their disease features resemble those of human disease [27,28]. The adjuvant models of myocarditis involve immunizations with complete Freund’s adjuvant (CFA) supplemented with myocarditogenic epitopes, such as Myhc-α 334–352, SERCA2a 971–990, ANT 21–40, β1AR 181–200, BCKDk 111–130, and cTNI 105–122 in A/J mice [29,30,31,32,33,34]; Myhc-α 614–643 in BALB/c mice [35]; and Myhc-α 1304–1320 in rats [30,31,32,33,34,36]. Although most of these disease models involve the mediation of CD4 T cells and/or antibodies, the role of CD8 T cells is unclear, and it has been a challenge to ascertain the antigen specificity of cardiac-reactive T cells in the causation of myocarditis for routine investigations. To address this issue, we generated T cell receptor (TCR) transgenic mice specific to Myhc-α 334–352, leading us to note the expression of Myhc-α 334–352-specific TCRs in both CD4 and CD8 T cells, providing opportunities to investigate the roles of both cell types in a single transgenic system.

## 2. Materials and Methods

### 2.1. Mice

Six-to-eight-week-old male and female C57BL/6 mice were obtained from The Jackson Laboratory (Bar Harbor, ME, USA). The animals were maintained based on the approved animal protocol guidelines of the University of Nebraska-Lincoln, Lincoln, NE, USA.

### 2.2. Generation of TCR Transgenic (Tg) Mice

To generate TCR-α and TCR-β constructs, we generated T cell hybridomas using IA^k^ dextramers for Myhc-α 334–352, as we have described previously [37]. cDNA was synthesized using the total RNA extracted from the Myhc-α 334–352 hybridoma; complete rearranged mRNA sequences of TCR-α and TCR-β were obtained using the Vα 5′rapid amplification of cDNA ends (RACE) and Vβ 5′RACE reverse primers. Essentially, the primers were designed in the constant regions of TCR to yield amplicons spanning the entire lengths of the rearranged leader (L), variable (V), and joining (J) regions of TCR-α and the L, V, diversity (D), and J regions of TCR-β. After ascertaining their sequences, forward primers were designed in the L sequence by incorporating the XmaI restriction enzyme (RE) site for TCR-α and the XhoI RE site for TCR-β. Similarly, reverse primers were designed from the intronic sequences that follow the Jα and Jβ by incorporating the NotI RE site for TCR-α and the SacII RE site for TCR-β. Using these primers, complete rearranged DNA sequences were obtained from the genomic DNA of the hybridomas. After cloning the amplicons into the intermediate vector (pAM2314, Addgene, Boston, MA, USA) and verifying the sequences, the rearranged genomic DNAs of TCR-α and TCR-β were cloned into the pTα and pTβ cassette vectors, respectively (a kind gift from Christophe Benoist and Diane Mathis, Harvard Medical School, Boston, MA, USA) [38,39,40]. Finally, the pTα and pTβ cassette vectors were cleaved with SalI and KpnI, respectively, and the resulting prokaryotic DNA-free TCR-α and TCR-β were used for injection into the pronuclei of the fertilized oocytes isolated from C57BL/6 mice (The Jackson Laboratory stock number 000664) at the Mouse Genome Engineering Core Facility, University of Nebraska-Medical Center, Omaha, NE. We bred TCR-α or TCR-β chains to obtain mice expressing both transgenes, as verified by qualitative polymerase chain reaction (PCR) (Table 1, top panel). The transgenic founder mice were bred with the C57BL/6 strain to establish the lines, and the lines were maintained in this strain background for all of the experiments. To determine the zygosity of transgenic mice, we adopted quantitative real-time (q) PCR using sequence-specific primers (Table 1, bottom panel). We donated these Tg mice to the Mutant Mouse Resource and Research Center for Cryopreservation (St. Louis, MO, USA) (MMRRC:068226-MU).

### 2.3. Genotyping

The Tg mice were screened by qualitative and quantitative PCR using genomic DNA extracted from mouse tails (DNA miniprep kit, BioPioneer Inc., San Diego, CA, USA). For qualitative PCR, two pairs of oligonucleotide primers, termed P2 and P3, were used to analyze the TCR α and β transgenes (Table 1). The PCR reactions in 20 µL volumes were carried out using 10 ng genomic DNA, OneTaq^®^ 2× Master Mix (New England Biolabs, Ipswich, MA, USA), and 10 pM of oligonucleotide primers. The reaction mixture was incubated at 94 °C for 3 min, followed by 32 cycles at 94 °C for 30 s, at 57.5 °C for 30 s, and at 68 °C for 25 s, and a final cycle at 68 °C for 5 min in CFX96^TM^ Real-Time System (Bio-Rad, Hercules, CA, USA). Agarose gel electrophoresis was used to separate the PCR products, which were visualized using ChemiDoc^TM^ MP Imaging System (Bio-Rad). For qPCR, a pair of fluorescent dye-labeled oligonucleotide primers (Table 1) was used to identify the TCR α and β transgenes using apolipoprotein B (ApoB) as an internal positive control (IPC). The reactions were performed in 15 µL volumes using 10 ng genomic DNA, OneTaq^®^ PrimeTime™ One-Step RT-qPCR Master Mix (Integrated DNA Technologies, Coralville, IA, USA), and PrimeTime^®^ qPCR assays (Integrated DNA Technologies) containing 500 nM primer and 250 nM probe. The mixtures were first incubated at 95 °C for 3 min, followed by 39 cycles at 95 °C for 5 s and 60 °C for 30 s to capture real-time amplification curves. The transgene expression was normalized to IPC using the 2^−(∆∆Ct)^ method, as reported previously [41].

### 2.4. Peptide Synthesis

Myhc-α 334–352 (DSAFDVLSFTAEEKAGVYK) and ovalbumin (OVA) 323–339 (ISQAVHAAHAEINEAGR) were synthesized and purified (>90%) by high-performance liquid chromatography and mass spectroscopy (Genscript, Piscataway, NJ, USA). After being dissolved in 1× phosphate-buffered saline, the peptides were aliquoted and stored at −20 °C.

### 2.5. Immunization Procedures

Myhc-α 334–352 immunizations involved peptide/CFA emulsions containing *Mycobacterium tuberculosis* H37RA extract (Difco Laboratories, Detroit, MI, USA) with a final concentration of 5 mg/mL. The emulsions were administered subcutaneously (100 µg/mouse) in the inguinal and sternum regions on days 0 and 7. In some experiments, animals received emulsions in the left and right shoulders and on the right and left sides of the hip regions in split doses. Additionally, pertussis toxin (List Biological Laboratories, Campbell, CA, USA) was administered to animals intraperitoneally (100 ng/mouse) on days 0 and 2 after the first immunization.

### 2.6. IA^b^ Binding Assay

To determine the binding affinity of Myhc-α 334–352 to IA^b^, soluble IA^b^ molecules were expressed similarly to the IA^k^ molecule [33,42]. Briefly, IA^b^ constructs were designed to contain the extracellular domains of IA^b^-α and IA^b^-β [43]. The IA^b^-β construct consisted of the class II-associated invariant chain peptide (CLIP) 88–102 (VSQMRMATPLLMRPM) and thrombin site to release the CLIP [44]. After expressing the IA^b^ molecules in baculovirus using Sf9 cells and purifying on an anti-IA^b^ affinity column (clone, M5/114) (BioXCell, West Lebanon, NH, USA), they were cleaved with thrombin to obtain empty IA^b^ molecules. Cocktails of reaction mixtures contained thrombin-cleaved IA^b^ (0.35 µg), competitor peptide Myhc-α 334–352 (0.00001 µM to 100 µM), and biotinylated OVA 323–339 (50 µM) as a reference peptide in a buffer with a composition of 50 mM sodium phosphate of pH 7.0, 100 mM sodium chloride, 1 mM ethylenediaminetetraacetic acid, and 1× protease inhibitor (Sigma Aldrich, St. Louis, MO, USA). The mixtures were incubated overnight at room temperature (RT). In addition, anti-IA^b^ (10 µg/mL) was coated onto white fluorescence 96-well plates (Nunc, Rochester, NY, USA) in 0.2 M sodium phosphate buffer, pH 6.8, overnight, followed by washing and blocking with 2% casein for 2 h at 37 °C. The reaction mixtures described above were then added after washing and incubated at RT (1 h) and washed. Europium-labeled streptavidin was added (1 µg/mL) in dissociation-enhanced lanthanide fluorescence immunoassay (DELFIA^®^) buffer and then the DELFIA^®^-enhancement solution (Perkin Elmer, Waltham, MA, USA). Fluorescence intensities were measured at wavelengths of 340/615 nm using a VICTOR^®^ Multilabel Plate Reader (Perkin Elmer), and IC_50_ values were then determined [33,42]. Under similar conditions, we performed the IA^k^ binding assay for Myhc-α 334–352 (positive control) as we have described previously [33].

### 2.7. Proliferation Assay

Spleens and draining lymph nodes were collected and single-cell suspensions termed lymphocytes were prepared. Cells were stimulated with Myhc-α 334–352 or OVA 323–339 (0–100 µg/mL) at a density of 5 × 10^6^ cells/mL for two days in growth medium containing RPMI-1640 supplemented with 10% fetal calf serum (HyClone, Logan, UT, USA), 4 mM L-glutamine (Gibco, Billings, MT, USA), 1 mM sodium pyruvate, 1× each of non-essential amino acids, a vitamin mixture, and 100 U/mL penicillin–streptomycin (Lonza, Basel, Switzerland). After pulsing with tritiated [^3^H] thymidine (1 µCi per well; Moravek Inc., Brea, CA, USA) for 16 h, proliferative responses were measured as counts per minute (CPM) using a Wallac liquid scintillation counter (Perkin Elmer).

### 2.8. Determination of Antigen Specificity of Myhc-α 334–352-Reactive T Cells by IA^b^ MHC Class II Dextramer Staining 

To generate IA^b^/Myhc-α 334–352 dextramers, IA^b^/CLIP precursors were prepared as described above. After biotinylation, IA^b^ molecules were treated with thrombin and CLIP was released. Myhc-α 334–352 was then loaded onto empty IA^b^ molecules by peptide-exchange reactions [33]. The dextramers were generated by incubating Myhc-α 334–352-exchanged IA^b^ molecules with fluorochrome-labeled dextran backbone [33,45]. We used IA^s^/Theiler’s murine encephalomyelitis (TEMV) 70–86 dextramers as controls [37,39]. Lymphocytes obtained from mice immunized with Myhc-α 334–352 were stimulated with Myhc-α 334–352 (50 µg/mL) for two days and maintained in growth medium containing interleukin (IL)-2 in order to determine the frequency of antigen-specific T cells. Viable lymphoblasts were harvested by Ficoll–Hypaque density gradient centrifugation on day 5 (MP Biomedicals, Santa Ana, CA, USA), and the cells were kept in growth medium containing IL-2. Dextramer staining was carried out on day 9 using IA^b^/dextramers, anti-CD4, and 7-aminoactinomycin D (7-AAD; Invitrogen, Carlsbad, CA, USA), as previously described [39]. After cells were acquired by flow cytometry (FACSCalibur™, BD Biosciences, San Jose, CA, USA), percentages of dextramer^+^ cells were determined in the 7-AAD^−^ (live) CD4^+^ subset using FlowJo software v10.9 (Tree Star, Ashland, OR, USA).

### 2.9. Immunophenotyping by Flow Cytometry

Splenocytes and thymocytes were stained for the expression of various markers using the antibodies indicated below, with clone identifiers for each indicated in parentheses: CD3 (17A2), CD4 (GK1.5), CD8 (YTS156.7.7), CD25 (PC61), CD44 (IM7), CD62L (MEL-14), CD69 (H1.2F3), FOXP3 (MF-14) (all from BioLegend, San Diego, CA, USA), and Vβ4 (marker of TCR-β transgene) (KT4, BD Biosciences). After incubating the cells in the dark on ice for 15 min and washing twice, the cells were acquired by flow cytometry (FACSCalibur™) and analyzed using FlowJo software v10.9.

### 2.10. Bulk TCR Sequencing

To confirm that CD4 and CD8 T cells from Tg mice express a common TCR, we sorted both cell types by flow cytometer-based cell sorting within the CD3^+^ subset using a cocktail of CD3, CD4, CD8, and 7-AAD. After extracting total RNA from the respective subsets, samples were subjected to bulk TCR sequencing using the mouse switching mechanism at the 5′end of the RNA template Mouse TCR α/β profiling kit (Takara Bio USA, San Jose, CA, USA). In brief, 5′RACE involved the capture of the entire V(D)J variable regions of TCR transcripts followed by semi-nested PCR to obtain TCR-α and -β chains. Libraries were prepared and sequenced using MiSeq (Illumina, Inc., San Diego, CA, USA) with a 2 * 300 read length, and the alignment and assembly of bulk TCR sequencing data were analyzed using MiXCR v2.1.5 (Takara Bio USA).

### 2.11. Cytokine Analysis

Lymphocytes were obtained from wild-type (Wt) or Tg mice immunized with or without Myhc-α 334–352. Where indicated, cells were restimulated with anti-CD3 (clone: 145–2C11, 2.5 µg/mL), Myhc-α 334–352, or OVA 323–339 (10–100 µg/mL), and the culture supernatants were collected. Cytokines were analyzed using the LEGENDplex Murine Th cytokine Panel (12-plex or 13-plex; BioLegend). The panel consisted of IFN-γ, IL-2, IL-5, IL-13, IL-9, IL-17A, IL-17F, IL-22, IL-6, tumor necrosis factor (TNF)-α, IL-21, and IL-10. The lyophilized cytokine standard mix was serially diluted to obtain the standard curve. After addition of the capture beads/cytokine antibody conjugates, detection antibodies and streptavidin–phycoerythrin reagents were added to the diluted standards and test samples. Beads were acquired by flow cytometry (FACSCalibur™), and concentrations of cytokines were determined using the LEGENDplex™ data analysis software suite (BioLegend). For measurement of IL-4, a mouse IL-4 enzyme-linked immunosorbent assay (ELISA) max standard set was used according to the manufacturer’s recommendations (BioLegend).

### 2.12. Histopathology

After fixing hearts in 10% phosphate-buffered formalin, three cross-sectional levels of each heart were sectioned (5 µm) and stained with hematoxylin and eosin (H and E). Sections were examined by a board-certified pathologist for inflammation and scored blinded to treatment as normal (0), mild multifocal (1–5 foci), moderate multifocal to coalescing (6–25 foci), and diffuse (26 or more foci) [46,47,48].

### 2.13. Statistical Analysis

All analyses, including the graphs, were performed using GraphPad Prism software v8.0 (GraphPad Software, Inc. La Jolla, CA, USA). Differences in the proliferative responses, IA^b^ binding assay, immunophenotyping, and cytokines were analyzed by Student’s *t*-test or two-way ANOVA. The T cell responses that had varied background levels in the Tg mice were scaled as described earlier [31,34]. The *p*-values ≤ 0.05 were considered significant.

## 3. Results

### 3.1. Myhc-α 334–352 Is a Promiscuous Immunogenic Epitope That Induces CD4 T Cell Response in Wt C57BL/6 Mice

**Figure 1:**
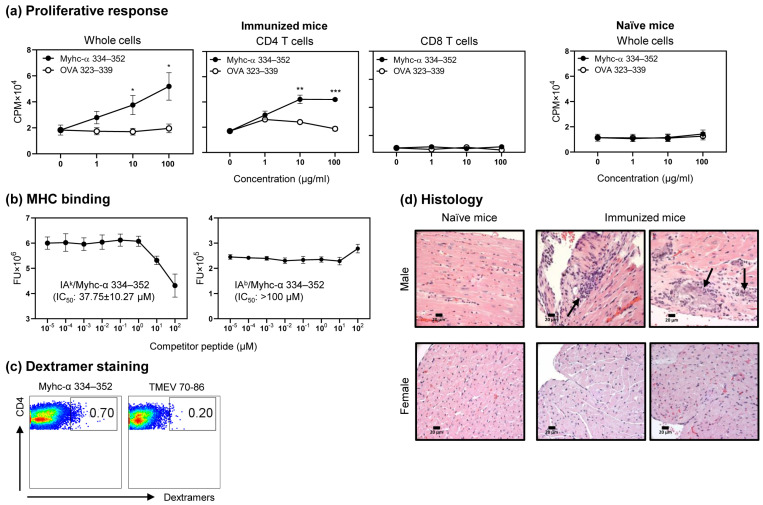
Myhc-α 334–352 is immunogenic in the Wt C57BL/6 mice and induces T cell responses. (**a**) Proliferative response. Groups of C57BL/6 mice were immunized with Myhc-α 334–352 in CFA, animals were euthanized after ten days, and lymph nodes were harvested to obtain single-cell suspensions. Whole cells (**left panel**) or CD4 and CD8 T cells (**middle panels**) obtained by magnetic separation were stimulated for two days with peptides, and after pulsing with [^3^H] thymidine, proliferative responses were measured as CPM. Similar analysis was performed on whole cells obtained from naïve mice (**right panel**). Mean ± SEM values from six to eight mice for whole cells and three mice for CD4 and CD8 T cells each are shown. (**b**) MHC binding. After expressing soluble IA^k^ and IA^b^ monomers in a baculovirus system, the corresponding empty molecules were derived by cleaving CLIP using thrombin. The reaction mixtures consisting of thrombin-cleaved IA^k^ or IA^b^ monomers and competitor peptide Myhc-α 334–352 and biotinylated HEL 46–61 (reference peptide for IA^k^) or biotinylated OVA 323–339 (reference peptide for IA^b^) were prepared and added to microplates coated with anti-IA^k^ or anti-IA^b^. After washing, europium-labeled streptavidin was added, followed by DELFIA^®^-enhancer solution. Fluorescence intensities were measured and IC_50_ values were calculated. Mean ± SEM values from three to five individual experiments involving three replicates in each are shown. (**c**) Dextramer staining. Myhc-α 334–352-stimulated lymphocytes prepared from immunized animals were stained with the indicated dextramers, anti-CD4 and 7-AAD, and the dextramer^+^CD4^+^ cells were analyzed in the live (7-AAD^−^) population after acquiring the cells by flow cytometry. Flow cytometric plots representing three individual experiments, each involving three to four mice, are shown. (**d**) Histology. Groups of animals were immunized with or without Myhc-α 334–352, and at termination on day 21 postimmunization, hearts were evaluated for inflammatory changes by H and E staining. Arrows in the top, middle, and right panels indicate lymphocytic infiltrations and fibrosis of myocardium, respectively, as opposed to normal sections on the left and bottom panels. Original magnification, ×400 (bar = 20 µm). Representative data from five experiments involving three to seven mice in each are shown. Two-way ANOVA was used to determine significance between groups. * *p* < 0.05; ** *p* < 0.01; and *** *p* < 0.001.

The IA^k^ epitope, Myhc-α 334–352, is a well-characterized epitope of T cells that induces myocarditis in A/J mice [33,46,49]. The immunogenic epitopes can be promiscuous in that they can bind multiple MHC alleles. For example, OVA 323–339 could bind IA^d^, IA^b^ and IA^g7^, respectively, in BALB/c, C57BL/6, and NOD/ShiLtJ mice [50,51,52]. In our efforts to determine the immunogenicity of Myhc-α 334–352 in C57BL/6 mice, we noted that lymphocytes obtained from Myhc-α 334–352-immunized animals responded to Myhc-α 334–352 dose-dependently and not to the control (OVA 323–339), thus defining the specificity of response (Figure 1a, left panel; *p* ≤ 0.05). Furthermore, by using magnetically sorted CD4 and CD8 T cells, we verified that the response was specific only to CD4 T cells (Figure 1a, middle panels; *p* ≤ 0.01). Induction of T cell responses to Myhc-α 334–352 required antigen priming in vivo, since lymphocytes from the naïve mice did not respond to Myhc-α 334–352 (Figure 1a, right panel). We next examined the MHC-binding ability of Myhc-α 334–352 to IA^b^ molecules, which required us to produce soluble IA^b^ molecules [33,42,43,53]. In brief, we created IA^b^-α and IA^b^-β constructs, and, after expression and antibody affinity column purification, the IA^b^ monomers tethered with CLIP 88–102 were treated with thrombin to obtain empty IA^b^ molecules. We used the IA^b^ and IA^k^ molecules (control) in a fluorescence-based DELFIA^®^ assay to determine the IC_50_ values for Myhc-α 334–352, using OVA 323–339 and hen egg lysozyme (HEL) 46–61 as reference peptides for the respective alleles [34,54]. The analyses indicated that Myhc-α 334–352 was found to bind IA^k^ molecules with an IC_50_ value of 37.75 ± 10.27 µM, as expected (Figure 1b, left panel). Under similar conditions, the IC_50_ value obtained with the IA^b^ molecules was more than 100 µM (Figure 1b, right panel), leading us to conclude that Myhc-α 334–352 is a poor binder of the IA^b^ molecule. Nonetheless, the IA^b^/Myhc-α 334–352 dextramers stained the Myhc-α 334–352-stimulated cells antigen-specifically (Figure 1c). The dextramer analysis revealed staining of CD4 T cells with Myhc-α 334–352 dextramers, whereas staining with control dextramers (TMEV 70–86) was negligible. Taken together, the data indicate that Myhc-α 334–352, despite being a poor binder of the IA^b^ molecule, can promote T cell activation. Finally, by evaluating the hearts of Myhc-α 334–352-immunized animals, we noted mild myocarditis, as indicated by the detection of a few inflammatory foci. Such a tendency was noted more in males than in females in a dose range of 50 to 100 µg/mouse (Figure 1d), except that one female mouse showed moderate multifocal foci at a dose of 200 µg/mouse (Table S1). Since animals immunized with Myhc-α 334–352 showed good T cell responses but developed only mild myocarditis, this model could be used to investigate the resistance mechanisms of myocarditis.

### 3.2. Generation of Myhc-α 334–352 TCR Tg Mice

To generate the rearranged genomic TCR-α and -β constructs, we used Myhc-α 334–352-specific T cell hybridomas that involved the use of Myhc-α 334–352 dextramers, as we have described previously [37]. We selected the clone C33, which was functional, as indicated by proliferation assay (Figure S1a). After ascertaining the complete cDNA sequences of TCR-α and -β by 5′RACE PCRs, we deduced the rearranged complete TCR sequences using the genomic DNA, leading us to confirm the compositions of TCR chains as follows: α chain (V14-1*02/J42*01/C*01) and β chain (V2*01, also denoted as vβ4/D/J2-5*01/C2*01), with the nucleotide sequence of the D region being AGTTTGGG (Table 2). The respective genomic constructs cloned into the pTα and pTβ vectors, which were freed of the prokaryotic DNA, were injected into the pronuclei of C57BL/6 oocytes. The founder mouse lines yielded pups positive for either α or β, as evaluated by qualitative PCRs using sequence-specific primers (Table 1 and Figure S1b). Therefore, we bred mice expressing TCR-α or -β for four generations to expand their colonies, and by crossing them together, we obtained mice expressing both α and β chains. Additionally, with qPCR analysis (Table 1), we could identify the Tg mice as heterozygotes or homozygotes for each chain (Figure S1c). The surviving heterozygous mice expressing TCR-α and the heterozygous or homozygous mice for TCR-β did not display any abnormal phenotypes in their growth. Nevertheless, the homozygotes for TCR-α consistently exhibited neurological symptoms, such as head tremors or head tilting, and circling behavior. Such phenotypes have been described in transgenic animals and are likely caused by random integration of the TCR-α gene into a locus controlling development of the inner ear [55,56]. Regardless of zygosity, we consistently noted that the litter sizes of Tg mice varied between two and five pups. Furthermore, cannibalism was often noted, and as a result, we could not obtain a large number of surviving Tg mice and their littermates. Therefore, whenever necessary, we opted to use the Wt mice and designated both non-Tg littermates and Wt mice as Wt controls for the transgenic mice during experimentation.

### 3.3. The Tg Mice Express Myhc-α 334–352-Specific TCR on Both CD4 and CD8 T Cells

**Figure 2:**
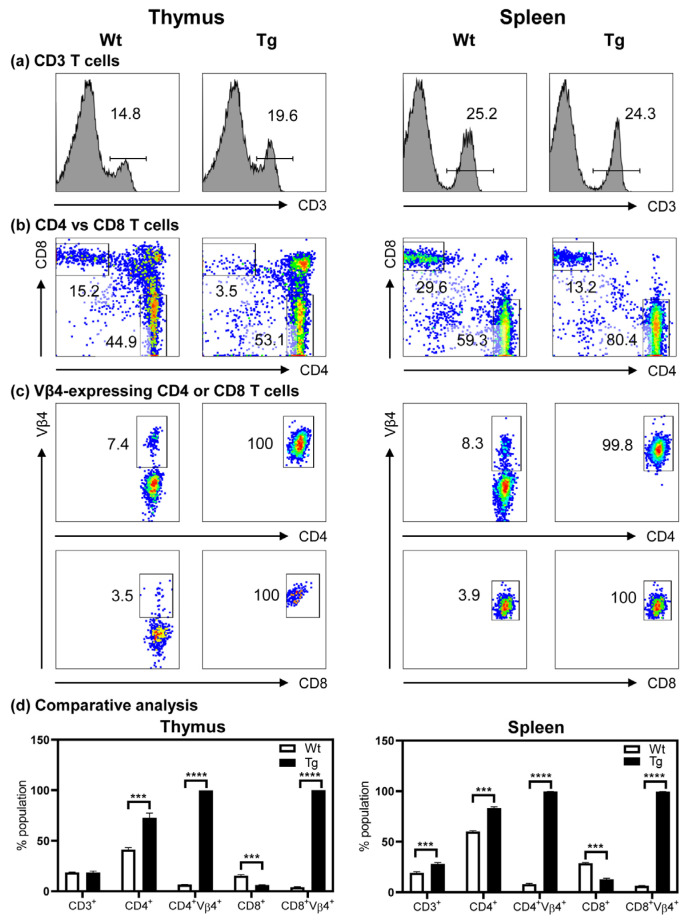
Myhc-α 334–352-specific TCRs are expressed in both CD4 and CD8 T cells in Tg mice. Cell suspensions were prepared from thymi (**left panels**) and spleens (**right panels**) of 6- to 8-week-old Wt and Tg mice. Cells were stained with anti-CD3, anti-CD4, anti-CD8, and anti-TCR vβ4, and after acquisition by flow cytometry, percentages of each subset were determined. Panels (**a**–**d**) represent the flow cytometric plots of CD3 T cells, CD4 and CD8 T cells within the CD3^+^ T cell subset, vβ4 expression in the CD4 and CD8 T cell subsets, and a comparative analysis for a group of three mice (mean ± SEM), respectively. Student’s *t* test was used to determine significance between groups. *** *p* < 0.001 and **** *p* < 0.0001.

We first evaluated the composition of T cell subsets in the thymi and spleens of Tg mice and compared it with that of Wt mice. The analyses revealed similar proportions of CD3^+^ T cells in both groups but with a marginal increase in the spleens of Tg mice (Figure 2a). Whereas the proportions of CD4 and CD8 double positive cells were low in the thymi and spleens of the Tg mice, no significant differences were noted in the double negative subset (CD4^−^CD8^−^) in the thymi of the Tg mice but were reduced in the spleens (Figure S2). Evaluation of the proportions of CD4 and CD8 T cells indicated that T cells in both the thymi and the spleens were skewed towards CD4 (~1.5 fold), with a corresponding decrease in the proportions of CD8 T cells (~2.0 fold) (Figure 2b). Third, the use of clonotypic TCR vβ4 antibody permitted us to analyze the expression of TCR-β. We unexpectedly noted that both CD4 and CD8 T cells expressed TCR-vβ4 in both the thymi and the spleens, suggesting that the Myhc-α 334–352-specific CD4 and CD8 T cells were positively selected in the thymi (Figure 2c). We also verified that both cell types in the lymph nodes expressed TCR-vβ4. We further confirmed the expression of TCR to be common for both CD4 and CD8 T cells by subjecting the RNA extracted from the corresponding cell types sorted by flow cytometry to bulk TCR sequencing. These analyses revealed the VJ and VDJ compositions to be similar for TCR-α and TCR-β, respectively (Table S2: For raw data, see spreadsheets 1 and 2 for CD4 and CD8 T cells, respectively; and Table 2). Additionally, the amino acid sequences of CDR1, CDR2, and CDR3 were similar (Table 2). Further analysis for activation markers (CD69 and CD25) and memory T cell populations [57] as determined by the expression of CD62L and CD44 revealed no significant variations, except that the proportions of CD62L^+^CD44^+^ CD4 T cells were low in the Tg mice (Figure S3). Likewise, no significant variations were noted with the FoxP3 expression that occurred only in CD4 T cells of both Wt and Tg mice (Figure S3). Taken together, although the TCRs were expressed in both CD4 and CD8 T cells, the T cell selection was skewed towards CD4 T cells in the Tg mice (Figure 2d), which prompted us to conclude that the Tg T cells respond to antigens.

### 3.4. T Cells from Naïve Tg Mice Remain Tolerant, and They Do Not Develop Myocarditis Spontaneously

**Figure 3:**
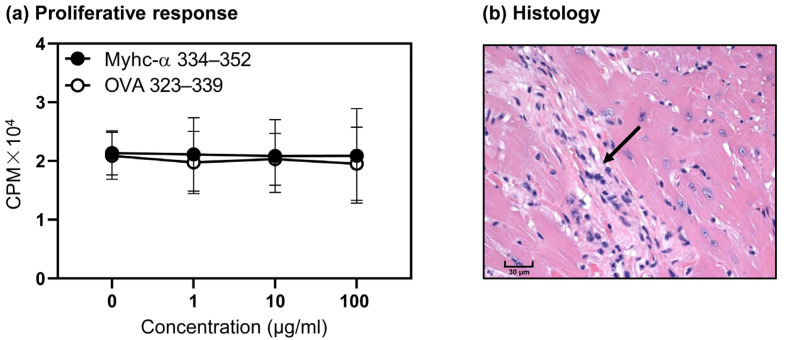
Tg T cells from naïve mice do not respond to Myhc-α 334–352. (**a**) Proliferative response. Lymphocytes from Tg mice were stimulated for two days with Myhc-α 334–352 or OVA 323–339 (control), and after pulsing for 16 h with [^3^H] thymidine, proliferative responses were measured as CPM. Mean ± SEM values representing three experiments are shown. (**b**) Histology. Heart sections prepared from naïve Tg mice were analyzed for histological changes by H and E staining. The arrow indicates fibrosis. Original magnification, ×400 (bar = 30 µm). The representative image of one animal from a group of nine is shown. Two-way ANOVA was used to determine significance between groups.

Using OVA 323–339 as a control peptide, we sought to determine the responsiveness of Tg T cells to Myhc-α 334–352 by proliferation assay. Contrary to expectations, the Tg T cells failed to respond to Myhc-α 334–352 at any of the doses tested (Figure 3a). We also confirmed that exposure to lipopolysaccharide during in vitro stimulation of Tg T cells with Myhc-α 334–352 did not promote T cell activation, ruling out a defect, if any, in the functionality of the antigen-presenting cells. By evaluating the hearts of naïve mice in the age range up to 10 months for histological changes, we noted fibrosis in only one mouse (1/9, 11.1%) (Figure 3b). The data point to the possibility that the Tg T cells might have acquired tolerance in the thymus or in the periphery. 

### 3.5. Immunization with Myhc-α 334–352 Breaks Tolerance in Tg Mice, and Immunized Animals Develop Myocarditis Albeit with Low Severity

**Figure 4:**
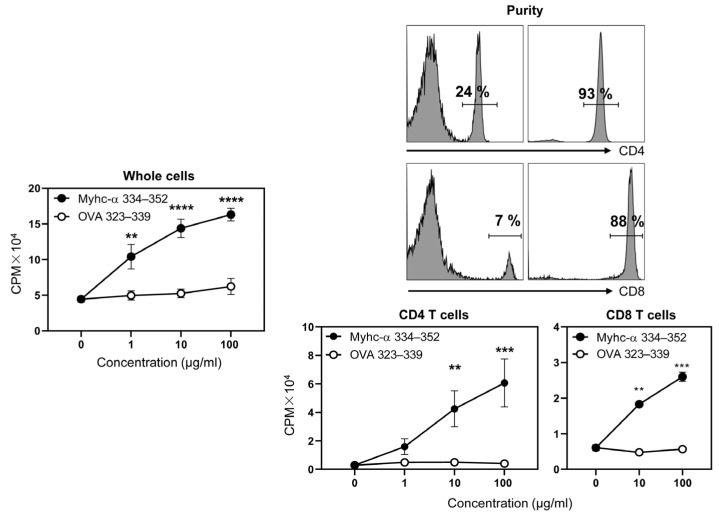
T cells from Myhc-α 334–352-immunized Tg mice respond to the peptide antigen-specifically. Tg mice were immunized with Myhc-α 334–352, and on day 21 at termination, lymphocytes from the immunized animals were stimulated with or without Myhc-α 334–352 and OVA 323–339 (control). After two days, whole cells were pulsed with [^3^H] thymidine for 16 h, the incorporation of which was measured as CPM (**left panel**). To determine the proliferative responses of CD4 and CD8 T cells, the corresponding subsets were sorted by magnetic separation (**top right panels**), and their ability to respond to Myhc-α 334–352 was evaluated as above using OVA 323–339 as the control (**bottom right panels**). Mean ± SEM values representing the data obtained from four mice for whole cells and CD4 T cells and three mice for CD8 T cells are shown. Two-way ANOVA was used to determine significance between groups. ** *p* < 0.01, *** *p* < 0.001, and **** *p* < 0.0001.

**Figure 5:**
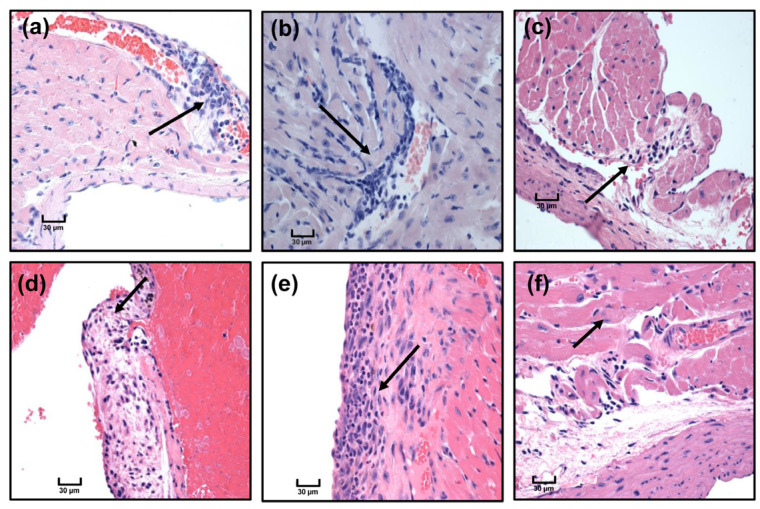
Tg mice develop mild myocarditis in response to Myhc-α 334–352 immunization. Tg mice were immunized twice with Myhc-α 334–352, and after 21 days, the animals were euthanized to collect the hearts for histological evaluation by H and E staining. Arrows indicate mononuclear cells (MNCs) in the epicardial vessel (**a**), MNCs beneath the endocardium (**b**), lymphocytes and neutrophils on the endocardial surface (**c**), epicardial lymphocytes (**d**), small valvular thickening with lymphocytes (**e**), and a few pericardial lymphocytes (**f**). Original magnification, ×400 (bar = 30 µm). Representative images are shown from a group of five mice.

To verify whether CFA/Myhc-α 334–352 breaks T cell tolerance in the Tg mice, we adopted the protocol involving the use of peptide emulsions twice with an interval of one week and three weeks later examined T cell proliferative responses [46,49,54]. The analysis revealed that the lymphocytes from immunized Tg mice proliferated dose-dependently, and the responses were specific to Myhc-α 334–352 but not to the control (OVA 323–339) (Figure 4, left panel; *p* ≤ 0.01). We next purified CD4 and CD8 T cells from the immunized animals by magnetic separation to a purity of ~90% to determine their responses to Myhc-α 334–352 (Figure 4, top right panels). Although both cell types responded specifically to Myhc-α 334–352, the proliferative responses were high in CD4 T cells compared to in CD8 T cells (Figure 4, bottom right panels; CD4 T cells ~14.9-fold, *p* ≤ 0.01 and CD8 T cells ~4.5-fold, *p* ≤ 0.01). We also verified that one dose of immunization was sufficient to induce T cell response (Figure S4, left panel). Furthermore, immunization with CFA alone did not lead to proliferative responses to Myhc-α 334–352 (Figure S4, right panel), suggesting that antigen-priming in vivo is necessary to break tolerance in the Tg mice. We next verified inflammatory changes in the hearts of the immunized mice, leading us to note only case of mild myocarditis in 5 out of 12 (~42%) animals examined (Figure 5). Furthermore, the infiltrates were present mostly in the epicardium, endocardium, or pericardium, suggesting that additional factors may be critical to inducing severe myocarditis in C57BL/6 mice.

### 3.6. Tg T Cells from Immunized Mice Preferentially Produce T Helper (Th)1 and Th17 Cytokines

**Figure 6:**
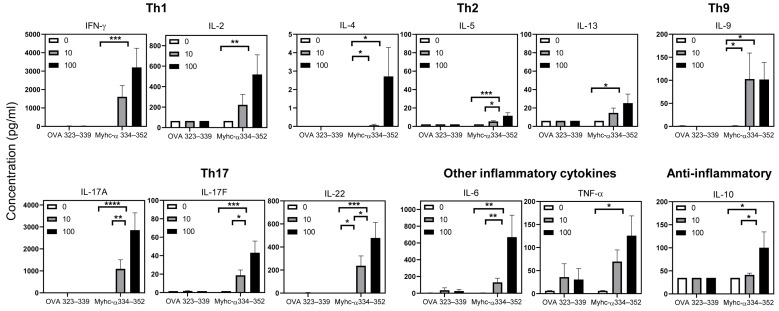
Tg T cells produce mainly Th1 and Th17 cytokines. Lymphocytes were prepared from Tg mice immunized twice with Myhc-α 334–352 on day 21 post-immunizations. Cells were stimulated with or without Myhc-α 334–352 or OVA 323–339 (control) at the indicated concentrations for two days, and the culture supernatants were evaluated by cytokine bead array analysis except for IL-4, which was measured by ELISA. Mean ± SEM values representing four individual experiments are shown. 0, medium control. Two-way ANOVA was used to determine the significance between groups. * *p* < 0.05, ** *p* < 0.01, *** *p* < 0.001 and **** *p* <0.0001.

To determine the basis for the lack of severe myocarditis in immunized Tg mice, we reasoned that Tg T cells might not be producing the typical inflammatory cytokines. To that end, we first examined the potential ability of Tg T cells to produce cytokines by stimulating the splenocytes from naïve Tg mice with anti-CD3 and compared the profiles with the Wt mice (Figure S5). We noted that the T cell responses from the Tg mice were relatively higher than those of Wt mice (Figure S6). The cytokine panel included Th1 (IL-2 and IFN-γ), Th2 (IL-4, IL-5, and IL-13), Th9 (IL-9), Th17 (IL-17A, IL-17F, and IL-22), and other inflammatory cytokines (IL-6, TNF-α, and IL-21), including anti-inflammatory cytokine IL-10. We noted that the Tg T cells produced mainly Th17 cytokines followed by TNF- α and to some degree IL-22 compared with Wt mice, but IL-4 was low (Figure S5, *p* ≤ 0.05). By extending these observations to the immunized Tg mice, it was evident that Myhc-α 334–352-primed Tg T cells produced mainly IFN-γ (317- to 633-fold, *p* ≤ 0.001), IL-17A (888- to 2323-fold, *p* ≤ 0.01), IL-22 (322- to 651-fold, *p* ≤ 0.05), and IL-6 (30- to 157-fold, *p* ≤ 0.01) in response to Myhc-α 334–352 stimulation dose-dependently compared to the medium controls (Figure 6). The lack of cytokine responses in cell cultures treated with the control (OVA 323–339) suggested specificity of cytokine production in Myhc-α 334–352-stimulated cells (Figure 6). Whereas other inflammatory cytokines (IL-9, ~75-fold, *p* ≤ 0.05; IL-17F, 12- to 28-fold, *p* ≤ 0.05; and TNF-α, 11- to 20-fold, *p* ≤ 0.05) were also marginally elevated, the depth of increased production of Th2 cytokines (IL-5, 2- to 5-fold; and IL-13, 2- to 4-fold), including IL-10 (~3-fold), in the Myhc-α 334–352-stimulated cultures was relatively low compared to that of Th1 and Th17 cytokines (Figure 6). Likewise, IL-4 was barely elevated (*p* ≤ 0.05). Thus, the lack of severe myocarditis in the Tg mice is unlikely to have been due to underproduction of pro-inflammatory cytokines. It is possible that soluble factors other than the above might have contributed to resistance to the development of myocarditis.

## 4. Discussion

In this report, we describe the generation of TCR Tg mice specific to Myhc-α 334–352, a well-characterized immunodominant epitope that induces autoimmune myocarditis in A/J mice [33,46,49,54]. Initially, we noted that Myhc-α 334–352 was found to be immunogenic in C57BL/6 mice, despite being a poor binder of IA^b^ molecules, while inducing only mild myocarditis. Similar observations have previously been reported for self-antigens, e.g., IA^u^/myelin basic protein (MBP) 1–20, or foreign antigens, e.g., IA^k^/*Zea mays* 159–174, IA^k^/*Magnetospirillum gryphiswaldense* 726–741, or IA^k^/*Cryptococcus neoformans* 93–108, and all of these were poor binders of the respective MHC molecules, yet they were immunogenic [54,58]. It may be that immunizations with potent adjuvants like CFA facilitate upregulation of MHC molecules with enhanced density on the surface of antigen-presenting cells (APCs), which may allow peptides to bind in clusters in order to induce T cell activation. In support of this notion, we noted that the IA^b^/Myhc-α 334–352 dextramers stained the T cells antigen-specifically. Nonetheless, since Myhc-α 334–352 is capable of inducing significant T cell responses in immunized C57BL/6 mice that resist development of myocarditis, as reported earlier [59], this model could be used to investigate myocarditis-resistant mechanisms in relation to other myocarditis-susceptible mouse strains.

In that direction, we sought to create TCR Tg mice specific to Myhc-α 334–352 and made a few observations. By characterizing the CD4 and CD8 T cell subsets, we observed skewing of CD4 to CD8 T cell ratios in the thymus and spleen, suggesting that T cell development favored the generation of CD4 T cells, as reported in other Tg systems [60,61]. However, expression of Myhc-α 334–352-specific TCRs in both CD4 and CD8 T cells was not expected. To our knowledge, our Tg model is only the second such example to have been reported, with the earlier example being 1C6 Tg mice specific to myelin oligodendrocyte glycoprotein (MOG) 35–55 [62]. We used the clonotypic vβ4 antibody to confirm the expression of TCR-β. Due to lack of a clonotypic antibody for vα14-1*02, we could not verify its expression using flow cytometry. Molecularly, however, we verified the compositions of TCR-α and -β transgenes by bulk TCR sequencing, and the analysis led us to note that CD4 and CD8 T Tg T cells expressed identical TCRs, including the compositions of CDRs. Such occurrences may be possible for antigens bearing the epitopes for both CD4 and CD8 T cells, and, indeed, both MOG 35–55 and Myhc-α 334–352 stimulate both cell types [49,62]. Specifically with respect to Myhc-α 334–352, we had earlier reported that this epitope possesses antigenic determinants for both CD4 and CD8 T cells in A/J mice, in which full-length Myhc-α 334–352 was shown to bind to MHC class II (IA^k^) alleles and its truncated version, Myhc-α 338–348, to bind to MHC class I (H-2^d^) alleles [49]. However, Myhc-α 338–348 was not exclusively specific to CD8 T cells since the peptide could stimulate both CD4 and CD8 T cells [49]. Similar cellular interactions might occur in vivo in C57BL/6 mice bearing the IA^b^ and H-2^b^ alleles. Thus, it is possible that the antigens bearing the determinants for both CD4 and CD8 T cells facilitate their selection in the thymus by binding to MHC class II and MHC class I alleles for the respective cell types. Detection of CD4 and CD8 T cells expressing Myhc-α 334–352-specific TCRs in the periphery may be supporting evidence for possible expression of Myhc-α in the thymi of C57BL/6 mice. Otherwise, no significant variations were noted in the expression of naïve or memory T cell markers, including FoxP3, the bona fide marker of Treg cells, whose expression was noted only in the CD4 T cells. Further, naïve Tg mice did not develop myocarditis spontaneously, which was not surprising since the Tg T cells from naïve mice do not react to Myhc-α 334–352, which prompted us to investigate the underlying mechanisms.

By immunizing the Tg mice, we first verified the T cell responses to Myhc-α 334–352. T cells from the immunized mice revealed significant responses to Myhc-α 334–352 in both CD4 and CD8 T cells. Although such responses could be obtained with a single immunization with peptide/CFA emulsions, CFA alone did not facilitate responses to Myhc-α 334–352. These findings suggest that the tolerance of Tg T cells in naïve mice could be broken but require priming with antigens in vivo. It is possible that unlike other mouse strains (A/J, BALB/cByJ, DBA/2J, A/J, and B10.D2) [63], APCs from C57BL/6 mice might not be displaying the Myhc epitopes for activating T cells in naïve mice. If this is the case, then why Tg T cells from naïve mice fail to respond to Myhc-α 334–352 in vitro is hard to explain. It may be that in vivo priming with Myhc-α 334–352 in combination with the activation signals received by CFA may be necessary to break tolerance to Myhc-α 334–352. Further, by evaluating heart sections from the immunized mice, we observed that about 42% of the animals developed myocarditis but with mild severity. The lack of disease in the immunized animals of other Tg systems, namely, MBP 1–20 and lymphocytic choriomeningitis virus glycoprotein, ascribed to changes in the environment, has been reported [64,65]. For example, the MBP Tg mice raised in rigorous specific pathogen-free (SPF) conditions did not develop experimental autoimmune encephalomyelitis [65,66]. We bred our Tg mice in the SPF facility free of known mouse pathogens, and whether the Myhc-α 334–352 Tg mice develop myocarditis in less rigorous conventional conditions remains to be tested. However, other factors inherent to the Tg T cells might also contribute to resistance to development of myocarditis.

To that end, we investigated cytokine production, initially verifying that Tg T cells have the potential to produce Th17 cytokines, in addition to IL-22 and TNF-α, through the stimulation of naïve T cells with anti-CD3, as analyzed by cytokine bead arrays. Similar analysis in Tg T cells obtained from immunized mice and stimulated with Myhc-α 334–352 revealed typical proinflammatory cytokines of Th1 and Th17 subsets that are known to be involved in the causation of autoimmune myocarditis [67,68], in addition to other inflammatory cytokines, namely, IL-6, IL-9, and TNF-α. Thus, insufficient production of inflammatory cytokines appears to be an unlikely cause for the lack of severe myocarditis noted in the Tg mice. Likewise, excessive production of Th2 cytokines (IL-4, IL-5, and IL-13), including anti-inflammatory cytokine IL-10, which could suppress the inflammatory functionalities of Th1 and Th17 cells [69,70,71,72], was not a factor; as such, production was significantly lower by several fold. Taken together, we believe that Tg T cells have the potential to produce myocarditis-inducing cytokines in naïve mice but are unable to do so because they remain tolerant. Under the conditions of a break in tolerance by CFA/Myhc-α 334–352 immunization, the Tg T cells can respond to antigens and induce myocarditis. However, the lack of severe disease in the immunized animals may suggest that soluble factors other than the cytokines we tested could contribute to resistance to myocarditis development in C57BL/6 mice.

In summary, we have described the generation of TCR Tg mice specific to Myhc-α 334–352 and shown that Tg T cells express TCRs common to both CD4 and CD8 T cells. The TCR transgenic models have been developed to study the roles of antigen-specific T cells for various self-antigens in autoimmunity research [73,74,75,76,77,78,79,80]. However, in myocarditis research, only one other model has been described for Myhc-α 614–629 that can develop myocarditis and DCM spontaneously on the BALB/c genetic background. However, in that system, the disease mediation involves only CD4 T cells [73]. Since lymphocytic myocarditis can involve both CD4 and CD8 T cells and their disease-inducing pathways are different, our model system can be used to further investigate their roles in a single transgenic system. In that direction, the roles of both CD4 and CD8 T cells as might occur in myocardial infarction [81,82] or chronic heart failure [83] and infections such as group B coxsackieviruses [84] and *Trypanosoma cruzi* [85] can be investigated in Tg mice. Since Tg C57BL/6 mice are highly resistant to the development of myocarditis, it may be possible to breed Tg mice with other genetically altered mice to understand the immune mechanisms of susceptibility to myocarditis. Despite producing significant amounts of Th1 and Th17 cytokines, the immunized animals fail to develop severe myocarditis, pointing to a possibility that soluble factors other than the typical inflammatory T cell cytokines might be critical. These aspects can be investigated by proteomic and transcriptomic analyses that may include single-cell RNA sequencing.

## Figures and Tables

**Table 1:** Primers used for genotyping the Myhc-α 334–352 Tg mice.

TCR Genes	Primers	Sequence (5′ to 3′)
Qualitative PCR		
α chain	P2 set:	
	Forward	GCAGCAGGAGAAACGTGAC
	Reverse	GCTTTGCATTGCTTCCTTCC
	P3 set:	
	Forward	CCTTGCACATCACAGACTC
	Reverse	TGAAGTGAGSTGGGGAATA
β chain	P2 set:	
	Forward	CGACCCAAAATTATCCAG
	Reverse	CAGTGCCTGGCCCAAAGTAC
	P3 set:	
	Forward	CCATTTAGACCTTCAGATCA
	Reverse	CCCAATCCCGCTGAGAAC
qPCR		
α chain	Forward	GTGCCTTTCCCGAAGGTTA
	Reverse	TCTCCTTGCACATCACAGAC
	Probe	56-FAM/TGCAGCAAG/ZEN/TGCGGAAGGAAG/3IABkFQ
β chain	Forward	TAACACGAGGAGCCGAGT
	Reverse	AGACCTTCAGATCACAGCTCTA
	Probe	5SUN/AGCAGCCAA/ZEN/AGTTTGGGCCAAG/3IABkFQ
Internal positive control	Forward	TCACCAGTCATTTCTGCTTTG
	Reverse	CACGTGGGCTCCAGATTT
	Probe	5Cy5/CCAATGGTC/TAO/GGGCACTGCTCAA/3lAbRQSp

**Table 2:** Compositions of Tg TCR-α and -β chains.

Cell Type	TCR Chains	Variable (V) Segment	Diversity (D) Segment	Joining (J) Segment	Constant Region	CDRs		
						CDR1	CDR2	CDR3
CD4 or CD8	α	TRAV14-1*02	-	TRAJ42*01	TRAC*01	DSTFNY	ISSVSDK	CAASAEGSNAKLTF
	β	TRBV2*01	D	TRBJ2-5*01	TRBC2*01	LGHNA	YSYQKL	CASSQSLGQDTQYF

## Data Availability

Not applicable.

## Supplementary Materials

The following supporting information can be downloaded at: https://www.mdpi.com/article/10.3390/cells12192346/s1, Figure S1: Proliferative response of T cell hybridoma and genotyping of Tg mice. After ascertaining the antigen specificity of the T cell hybridoma by proliferation assay (panel a), genomic DNA was extracted to prepare the TCR-α and TCR-β constructs to generate Tg mice from the T cell hybridoma. For genotyping, the genomic DNA extracted from tails was subjected to qualitative and qPCR, as shown in panels b and c, respectively. For qualitative PCR, two sets of primers (P2 and P3) were each used for TCR-α and TCR-β, and the PCR products were resolved on ethidium bromide-containing agarose (1.5%) gel electrophoresis; expected target sizes are shown (panel b). Homozygotes and heterozygotes were confirmed with qPCR, which involved the use of ApoB as a positive control, to which the amplifications of TCR-α and TCR-β transgenes were compared for quantitative analysis of transgenes (panel c). Figure S2: Comparison of double positive (CD4^+^CD8^+^) and double negative (CD4^−^CD8^−^) T cells in thymi and spleens from Wt and Tg mice. Cell suspensions were prepared from thymi (left panel) and spleens (right panel) of 6- to 8-week-old Wt and Tg mice. Cells were stained with anti-CD3 anti-CD4 and anti-CD8 and acquired by flow cytometry. The percentages of each subset were determined, and comparison was made between groups. Mean ± SEM values representing 3 mice per group are shown. Student’s *t* test was used to determine significance between groups. ** *p* < 0.01 and *** *p* < 0.001. Figure S3: Characterization of T cell subsets in Tg mice. Splenocytes from Wt and Tg mice were analyzed for expression of markers representing activation (CD69 and CD25), naïve, and memory T cells (CD62L and CD44) and FoxP3 by flow cytometry (top panels). Cells positive for each marker were analyzed in CD4 and CD8 T cell subsets, as shown in the bottom left and right panels, respectively. Student’s *t* test was used to determine significance between groups. ** *p* < 0.01. Figure S4: Tolerance of Tg T cells can be broken with a single immunization but not with CFA alone. Tg mice were immunized with a single dose of Myhc-α 334–352 in CFA (left panel) or CFA alone (right panel), and PT was administered on days 0 and 2. Three weeks later, lymphocytes were harvested at termination, and cells were stimulated with the indicated peptides for two days. After pulsing with [^3^H] thymidine for 16 h, proliferative responses were measured as CPM. Two-way ANOVA was used to determine the significance between groups. * *p* < 0.05 and ** *p* < 0.01. Figure S5: Tg T cells have the potential to produce inflammatory cytokines. Lymphocytes obtained from Wt and Tg mice were stimulated with anti-CD3 for three days, and the supernatants were analyzed by cytokine bead array analysis except for IL-4, which was measured by ELISA. Two-way ANOVA was used to determine the significance between groups. Mean ± SEM values representing the data obtained from five Wt and four Tg mice are shown. * *p* < 0.05, ** *p* < 0.01, and *** *p* < 0.001. Figure S6: Comparison of T cell responses to anti-CD3 stimulation between Wt and Tg mice. Cell suspensions were prepared from splenocytes harvested from the indicated groups of mice. After stimulating with anti-CD3 (0–2.5 µg/mL) for two days. After pulsing with [^3^H] thymidine for 16 h, proliferative responses were measured as CPM. Mean ± SEM values representing 2 to 3 mice per group are shown. Two-way ANOVA was used to determine the significance between groups. *** *p* <0.001. Table S1: Histological evaluation of myocarditis induced by Myhc-α 334–352 in the Wt C57BL/6 mice. Table S2: Raw data of bulk TCR sequencing.
