# Supplementary material for: Transgenic Mice Expressing Functional TCRs Specific to Cardiac Myhc-α 334–352 on Both CD4 and CD8 T Cells Are Resistant to the Development of Myocarditis on C57BL/6 Genetic Background
Source: Cells. 2023 Sep 25;12(19):2346. doi: 10.3390/cells12192346 (PMC10571761; doi:10.3390/cells12192346)
Supplement: Supplementary file 1 [file cells-12-02346-s001.zip › cells-2602643-supplementary.docx]

**Table S1.** Histological evaluation of myocarditis induced by Myhc-α 334–352 in the Wt C57BL/6 mice.

| **Sex** | **Dose**  **(µg)** | **Incidence**  **(%)** | **Inflammatory foci**  **(Mean ± SEM)** |
| --- | --- | --- | --- |
| Male | 50 | 4/7 (57.1) | 1.8 ± 0.3 |
|  | 100 | 4/13 (30.8) | 2.0 ± 0.4 |
|  | 200 | 1/8 (12.5) | 1.0 ± 0.0 |
| Female | 50 | 0/5 (0) | 0.0 ± 0.0 |
|  | 100 | 1/13 (7.7) | 21.0 ± 0.0 |
|  | 200 | 0/7 (0) | 0.0 ± 0.0 |


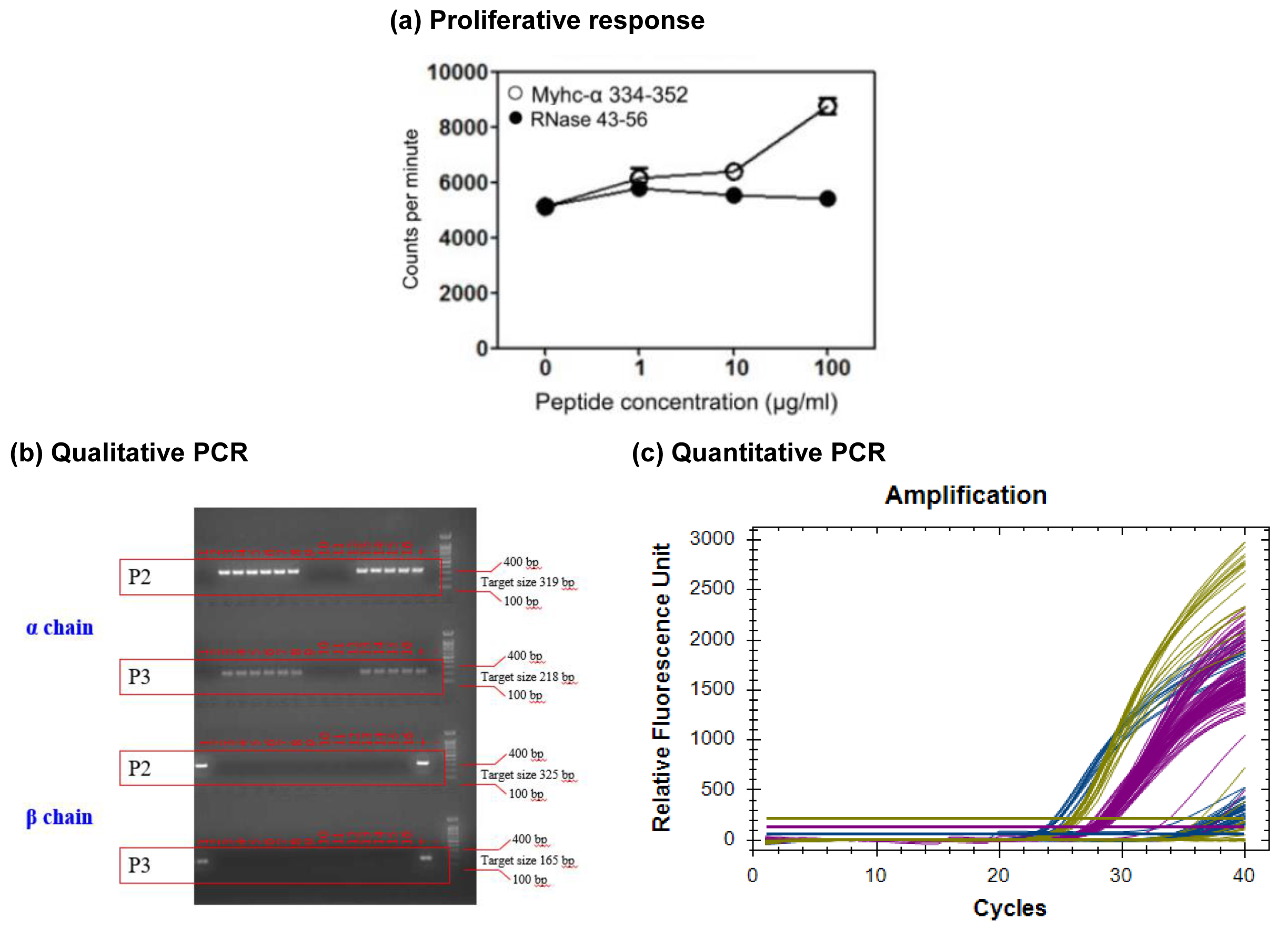


**Figure S1.** Proliferative response of T cell hybridoma and genotyping of Tg mice. After ascertaining the antigen specificity of the T cell hybridoma by proliferation assay (panel a), genomic DNA was extracted to prepare the TCR-α and TCR-β constructs to generate Tg mice from the T cell hybridoma. For genotyping, the genomic DNA extracted from tails was subjected to qualitative and qPCR, as shown in panels b and c, respectively. For qualitative PCR, two sets of primers (P2 and P3) were each used for TCR-α and TCR-β, and the PCR products were resolved on ethidium bromide-containing agarose (1.5%) gel electrophoresis; expected target sizes are shown (panel b). Homozygotes and heterozygotes were confirmed with qPCR, which involved the use of ApoB as a positive control, to which the amplifications of TCR-α and TCR-β transgenes were compared for quantitative analysis of transgenes (panel c).


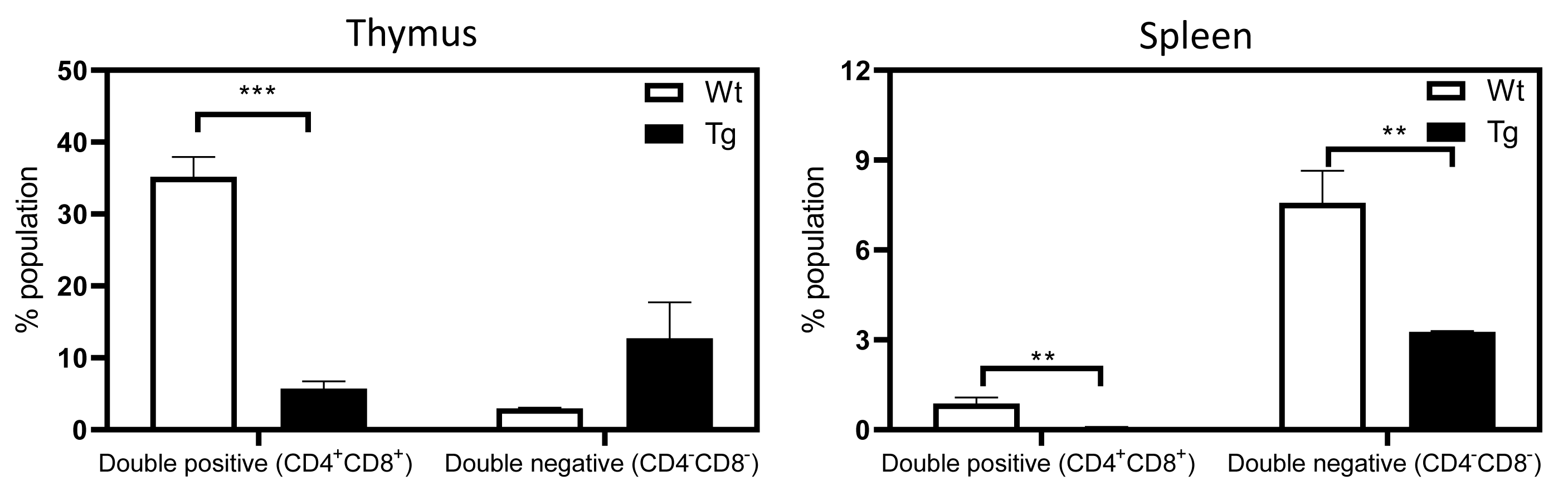


**Figure S2.** Comparison of double positive (CD4^+^CD8^+^) and double negative (CD4^-^CD8^-^) T cells in thymi and spleens from Wt and Tg mice. Cell suspensions were prepared from thymi (left panel) and spleens (right panel) of 6- to 8-week-old Wt and Tg mice. Cells were stained with anti-CD3 anti-CD4 and anti-CD8 and acquired by flow cytometry. The percentages of each subset were determined, and comparison was made between groups. Mean ± SEM values representing 3 mice per group are shown. Student’s t test was used to determine significance between groups. ** *p* < 0.01 and *** *p* < 0.001.


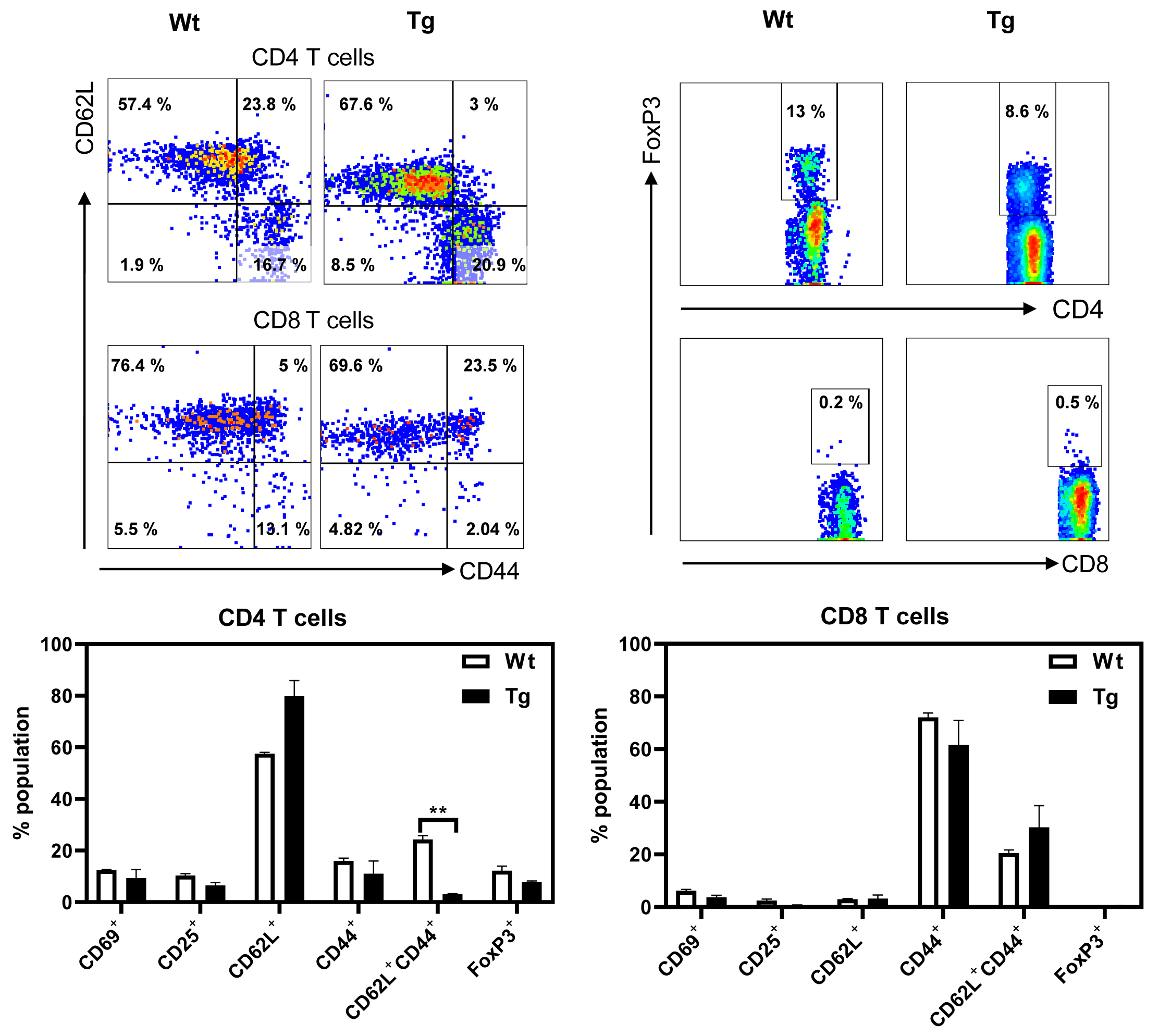


**Figure S3.** Characterization of T cell subsets in Tg mice. Splenocytes from Wt and Tg mice were analyzed for expression of markers representing activation (CD69 and CD25), naïve, and memory T cells (CD62L and CD44) and FoxP3 by flow cytometry (top panels). Cells positive for each marker were analyzed in CD4 and CD8 T cell subsets, as shown in the bottom left and right panels, respectively. Student’s t test was used to determine significance between groups. ** *p* < 0.01.


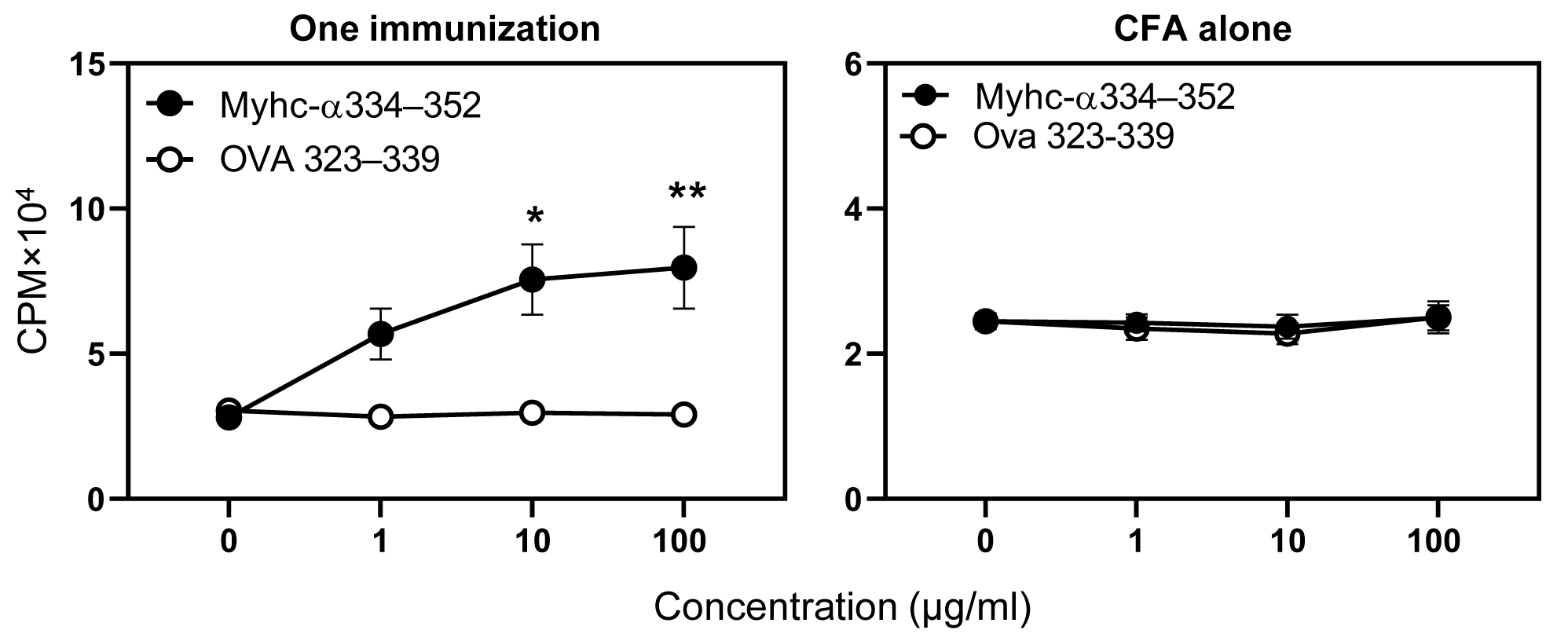


**Figure S4.** Tolerance of Tg T cells can be broken with a single immunization but not with CFA alone. Tg mice were immunized with a single dose of Myhc-α 334–352 in CFA (left panel) or CFA alone (right panel), and PT was administered on days 0 and 2. Three weeks later, lymphocytes were harvested at termination, and cells were stimulated with the indicated peptides for two days. After pulsing with [^3^H] thymidine for 16 h, proliferative responses were measured as CPM. Two-way ANOVA was used to determine the significance between groups. * *p* < 0.05 and ** *p* < 0.01.


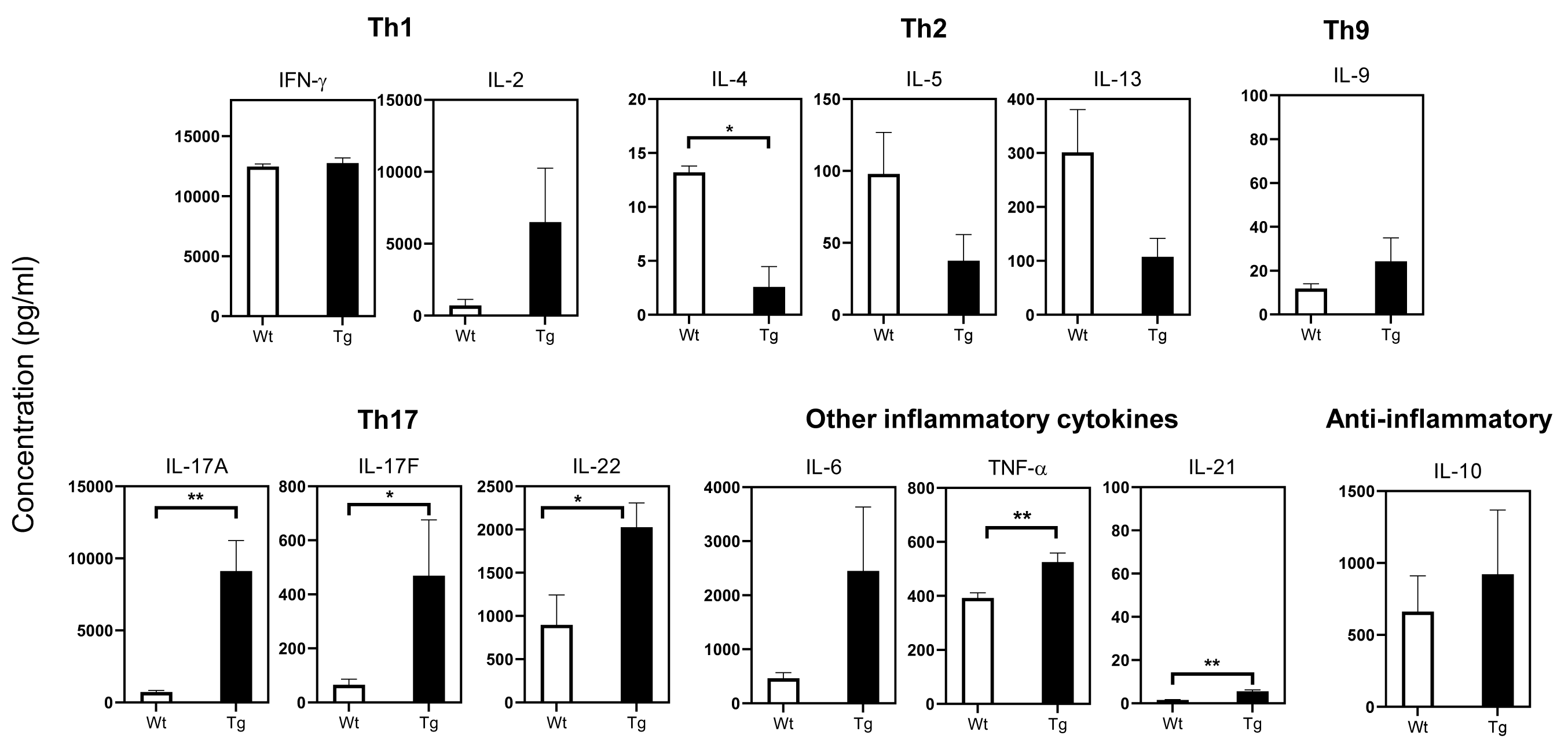


**Figure S5.** Tg T cells have the potential to produce inflammatory cytokines. Lymphocytes obtained from Wt and Tg mice were stimulated with anti-CD3 for three days, and the supernatants were analyzed by cytokine bead array analysis except for IL-4, which was measured by ELISA. Two-way ANOVA was used to determine the significance between groups. Mean ± SEM values representing the data obtained from five Wt and four Tg mice are shown. * *p* < 0.05, ** *p* < 0.01, and *** *p* < 0.001.


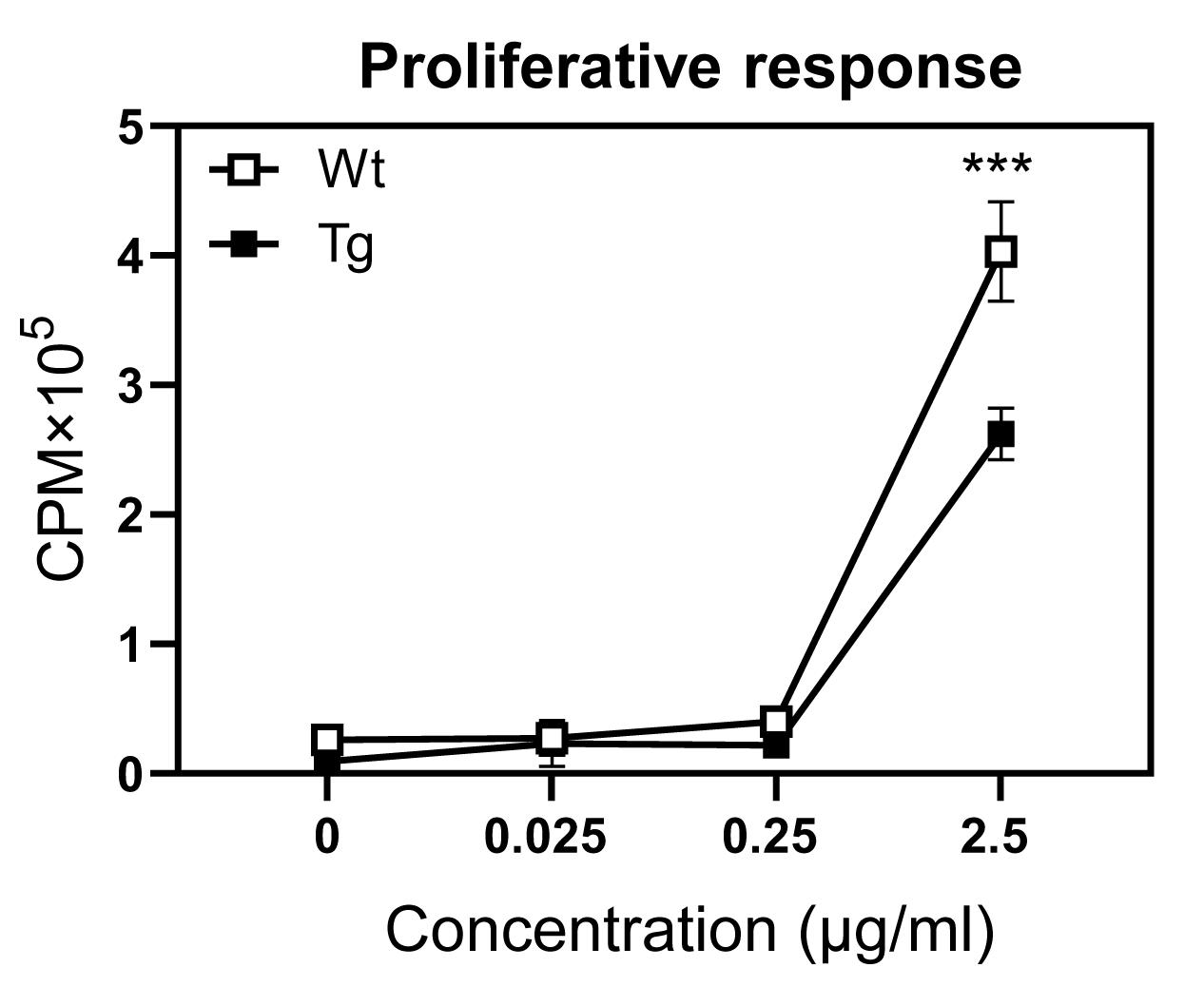


**Figure S6.** Comparison of T cell responses to anti-CD3 stimulation between Wt and Tg mice. Cell suspensions were prepared from splenocytes harvested from the indicated groups of mice. After stimulating with anti-CD3 (0–2.5 µg/ml) for two days. After pulsing with [^3^H] thymidine for 16 h, proliferative responses were measured as CPM. Mean ± SEM values representing 2 to 3 mice per group are shown. Two-way ANOVA was used to determine the significance between groups. *** *p* <0.001.
